# Respiratory Syncytial Virus Can Infect Basal Cells and Alter Human Airway Epithelial Differentiation

**DOI:** 10.1371/journal.pone.0102368

**Published:** 2014-07-17

**Authors:** B. David Persson, Aron B. Jaffe, Rachel Fearns, Henry Danahay

**Affiliations:** 1 Developmental and Molecular Pathways, Novartis Institutes for BioMedical Research, Cambridge, Massachusetts, United States of America; 2 Department of Microbiology, Boston University School of Medicine, Boston, Massachusetts, United States of America; 3 Respiratory Disease Area, Novartis Institutes for BioMedical Research, Horsham, United Kingdom; Lovelace Respiratory Research Institute, United States of America

## Abstract

Respiratory syncytial virus (RSV) is a major cause of morbidity and mortality worldwide, causing severe respiratory illness in infants and immune compromised patients. The ciliated cells of the human airway epithelium have been considered to be the exclusive target of RSV, although recent data have suggested that basal cells, the progenitors for the conducting airway epithelium, may also become infected *in vivo*. Using either mechanical or chemical injury models, we have demonstrated a robust RSV infection of p63^+^ basal cells in air-liquid interface (ALI) cultures of human bronchial epithelial cells. In addition, proliferating basal cells in 2D culture were also susceptible to RSV infection. We therefore tested the hypothesis that RSV infection of this progenitor cell would influence the differentiation status of the airway epithelium. RSV infection of basal cells on the day of seeding (MOI≤0.0001), resulted in the formation of an epithelium that showed a profound loss of ciliated cells and gain of secretory cells as assessed by acetylated α-tubulin and MUC5AC/MUC5B immunostaining, respectively. The mechanism driving the switch in epithelial phenotype is in part driven by the induced type I and type III interferon response that we demonstrate is triggered early following RSV infection. Neutralization of this response attenuates the RSV-induced loss of ciliated cells. Together, these data show that through infection of proliferating airway basal cells, RSV has the potential to influence the cellular composition of the airway epithelium. The resulting phenotype might be expected to contribute towards both the severity of acute infection, as well as to the longer-term consequences of viral exacerbations in patients with pre-existing respiratory diseases.

## Introduction

Human respiratory syncytial virus (RSV) infections are estimated to cause 64 million cases of respiratory disease worldwide leading to acute lower respiratory infections and 160,000 deaths annually [1], [2]. RSV has seasonal epidemiology causing annual epidemics and virtually 100% of children are infected before the age of two. Neonates, young children, immune-compromised adults and the elderly are especially prone to severe infection, resulting in bronchiolitis associated with epithelial necrosis, sloughing of the airway epithelium, edema and increased secretion of mucus. RSV infection is also known to frequently exacerbate existing pulmonary conditions such as chronic obstructive pulmonary disease (COPD) and asthma [3], [4], [5]. Exposure to RSV does not provide life-long lasting immunity allowing for recurrent infections through life [6], [7], and a greater understanding of the host response to this virus is expected to provide new insights into future therapy. To date, treatment of RSV disease is restricted to supportive care and prophylactic administration of palivizumab (Synagis), a monoclonal antibody directed to the fusion protein, for high-risk groups. No effective small molecule compounds or vaccines are currently available [8], [9].

The major site of RSV infection in the human lung is the pseudostratified epithelium that lines the conducting airway. This epithelium is composed of apical multi-ciliated and secretory (goblet) cells with the p63^+^ basal cell located directly beneath this layer. Elegant studies using well differentiated air-liquid interface (ALI) cultures of primary human bronchial epithelial cells (HBEC) have convincingly demonstrated that RSV has tropism for ciliated cells [10], [11], [12], [13], [14], [15]. However, *in vivo* work using an infant baboon model and a pre-term lamb model have also described the potential for the airway basal cell to become infected by RSV. These studies propose that virus-induced damage to the surface epithelium enables access of RSV to an otherwise inaccessible, non-ciliated cell-type [16], [17]. The identity of the infected, non-ciliated cell in these studies was not examined, but could potentially be a basal cell. Considering that respiratory diseases such as asthma and COPD can be associated with disrupted epithelial cell-cell junctions, impaired barrier function, and sloughing of the epithelium, basal cells might be reasonably expected to also be accessible to viruses such as RSV in patients with these pre-existing respiratory conditions [18], [19], [20], [21], [22]. The implications for infection of an airway basal cell are potentially widespread, especially in view of the key progenitor role it serves [23]. However, this remains a largely unexplored area, most likely because: 1) human airway basal cells in ‘steady-state’ ALI culture have been reported to not become infected by RSV, even after mechanical injury to the epithelium [11], and 2) human pathology studies largely implicate the ciliated cell as the major site of infection, and although infected non-ciliated cells have been described [24], basal cells have been considered to be resistant to RSV. It should however be considered that human pathology data are mostly restricted to pediatric cases [25], [26], [27]. To our knowledge, there are no pathology reports relating to RSV infection in adult patients with pre-existing conditions such as asthma or COPD, where epithelial barrier function can be chronically impaired. Furthermore, basal cells in a damaged epithelium will be required to be highly proliferative, that is in contrast to their slow turnover in the ‘steady-state’, healthy epithelium. The potential for RSV to infect basal cells in this highly proliferative state has not been explored. In view of the potential significance of an RSV infection of basal cells to human disease, recent *in vivo* data suggesting infection of basal cells together with outstanding questions regarding the identity of the infected non-ciliated cell, in human pathology studies, we have re-evaluated the basal cell tropism question in ALI cultures of HBEC.

Here we show that p63^+^, primary human airway basal cells from multiple donors can be readily infected by different strains of RSV. We found that infection of basal cells occurred in both a 2D cell culture system, as well as in well-differentiated, polarized cultures grown at ALI. Mechanical injury (scratch wound) or chemical damage (low Ca^2+^ disruption of adherens junctions and desmosomes) both resulted in exposure of basal cells to RSV and a subsequent infection. The consequence of infection of the basal cell is that the resultant epithelium had a reduced density of ciliated cells and a greater proportion of mucin-containing goblet cells. This switch in epithelial phenotype might be reasonably expected to be a normal protective effect if appropriately regulated in a normal healthy individual, but could equally contribute towards airway occlusion and impaired mucociliary clearance in a patient with a pre-existing respiratory disease.

Furthermore, we have found that a number of interferons are able to phenocopy the effect of RSV on basal cell fate determination and have extended these observations to highlight that the endogenous production of the type III interferon IL-29 following RSV infection at least partially drives the loss of ciliated phenotype. To our knowledge, these are the first data to outline a mechanism by which RSV infection may contribute to airway epithelial remodeling and therefore the pathology of a number of respiratory diseases. Future therapies designed to regulate basal cell fate determination in the lung following injury or infection could represent novel approaches to the treatment of viral diseases in an adult host suffering from pre-existing conditions such as asthma, cystic fibrosis and COPD.

## Results

### RSV infects human airway basal cells

**Figure 1:**
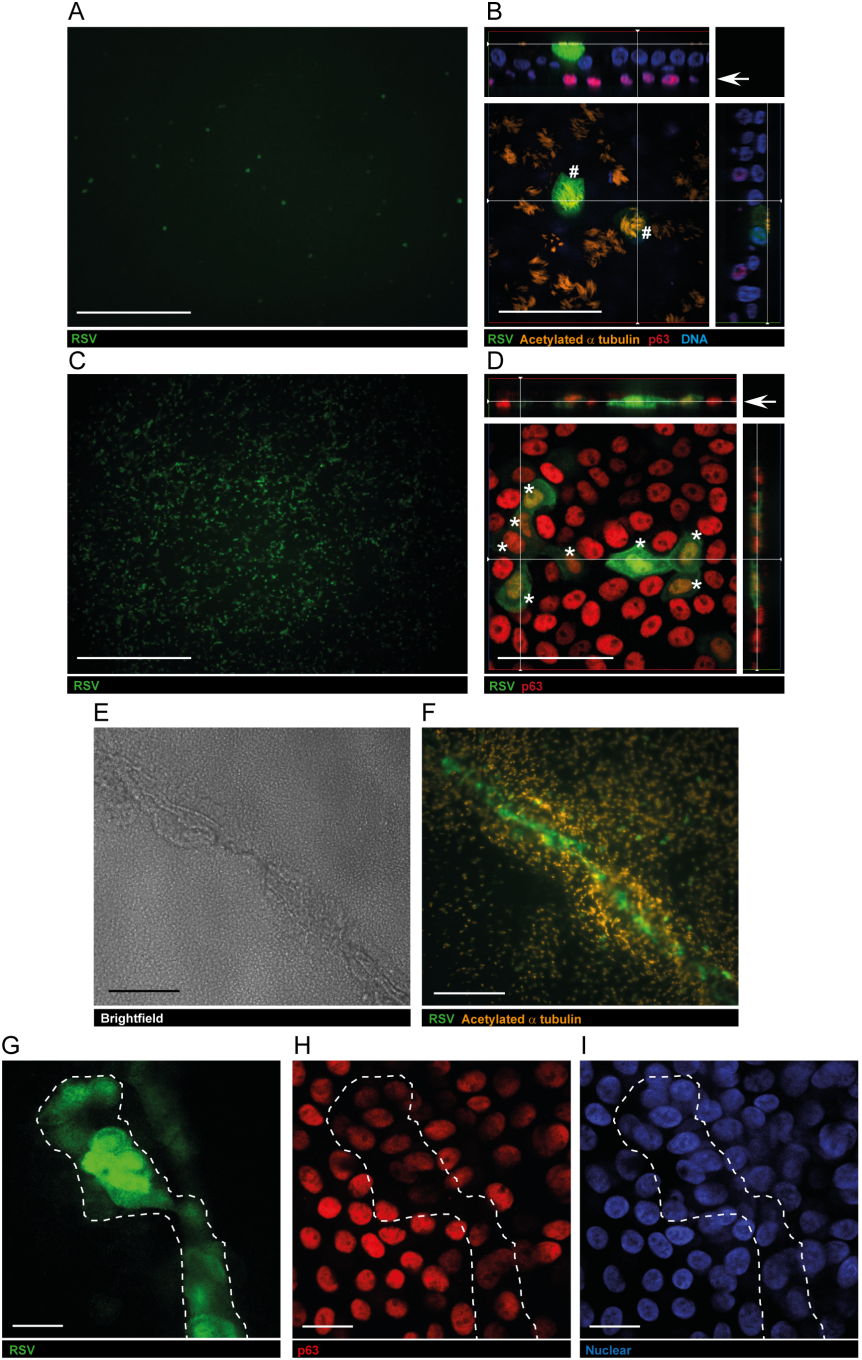
RSV infection of both multi-ciliated and airway basal cells. Apical exposure of HBEC to RSV-A2-GFP led to the infection of multi-ciliated cells. GFP^+^ (infected) cells were observed throughout the epithelium. These were identified as an acetylated α-tubulin^+^, ciliated cell (#, orange) by confocal imaging (A–B). Under these conditions, the sub-apical p63^+^ basal cells (red) did not become infected by RSV (B). Removal of the surface of the epithelium immediately before exposure to RSV resulted in numerous p63^+^GFP^+^ (*) cells 16 h later (C–D), consistent with infection of the airway basal cell. When well-differentiated cells were subjected to a mechanical scratch injury (E) followed by RSV infection multiple infected cells were clearly visible within the wound (F). A majority of the infected cells were determined as basal cells (p63^+^) by confocal microscopy (G–I). Presented are representative images from three independent experiments in two different donors. White bar indicates 1,000 µm in A, C, 50 um in B, D, 500 µm in E–F and 20 µm in G–I.

The experiments performed in these studies used well-differentiated cultures of the human airway epithelium that have been previously described by others [28], [29],[30]. This model utilizes p63^+^ primary human airway basal cells cultured on a permeable support. The cells first proliferate and form a confluent epithelium (days 0–7), while submerged in growth medium, and then differentiate over days 8–21 while exposed to an air-liquid interface (ALI). This model has been widely used to study the regulation of airway epithelial growth and repair and to investigate RSV infection of the human airway [11], [12], [13], [15]. When ALI cultures were exposed to recombinant RSV, engineered to express green fluorescent protein (RSV-A2-GFP) [31], [32] (150,000 pfu/Transwell), we observed a widespread infection of ciliated cells (Figure 1A, B), consistent with published work [11], [12], [15]. Confocal microscopy failed to identify any GFP^+^ infected cells in the basal region of the epithelium (Figure 1B), also consistent with previous reports [11], [13], [15]. To investigate whether an alteration in the integrity of the epithelial barrier would affect RSV tropism, we treated fully differentiated ALI cultures with a calcium-free medium using a protocol previously described to enable rhinovirus access to airway basal cells [33]. After a short exposure to calcium-free media, the supra-basal cell layer detached and could be easily removed, leaving a layer of predominantly p63^+^ basal cells. RSV-A2-GFP was added to the remaining monolayer of p63^+^ cells (150,000 pfu/Transwell) and at 16 hours post infection numerous dual positive GFP^+^p63^+^ cells were clearly evident across the insert (Figure 1C–D).

To test whether the infection of p63^+^ basal cells with RSV was an artifact of treatment with Ca^2+^-free media, we next mechanically injured the ALI cultures using the protocol described by Zhang et al. [11]. Intersecting thin scratches were made across the Transwell filter using a GUM stimulator, immediately after which 250,000 pfu of RV-A2-GFP was added. After a 30 minute exposure to RSV, the viral inoculum was removed and the cells cultured for a further 20 h. By 20 h after injury and infection, the scratch wound areas appeared to have covered over with epithelial cells (Figure 1E) and infected (GFP^+^) cells were clearly visible within the wound area (Figure 1F). Confocal imaging of the wound area defined the infected cells as dual positive p63^+^GFP^+^, indicating infection of the airway basal cells (Figure 1G–I). To quantify the infection of basal cells within the scratch injury we outlined the approximate area of the injury in 12 imaged z-stacks (similar as indicated in Figure 1G–I), from 2 independent experiments, and counted the total number of p63^+^ basal cells and GFP^+^ infected cells. In total, of 214 p63^+^ basal cells that were counted in the wound areas, 93 cells were RSV infected. In addition, we only found three infected cells within the marked scratch area that were neither p63^+^ nor ciliated. In undamaged areas of the insert, an extensive infection of ciliated cells was observed. Together the data highlighted that RSV can infect basal cells if it is able to gain access to the sub-apical region of the epithelium.

An observation made in the two ALI injury/infection studies above, was that the p63 levels appeared to be reduced in the GFP^+^ cells. To address whether RSV infection of basal cells could repress p63 protein expression, basal cells were seeded onto Transwell inserts and infected with RSV 3 h later. At 48 h after seeding (45 h post infection) cells were fixed and FACS sorted using the basal cell markers p63 and ITGA6 together with GFP as a marker of infected cells. We confirmed that 100% of infected cells were p63^+^ITGA6^+^ (Figure S1A–D) and that there was a significant reduction in the mean intensity of the p63 immunostaining (64.9±1.2% of uninfected control; p<0.002), consistent with observations made in the ALI studies.

### Basal cell infection sustains the epithelial spread of RSV

**Figure 2:**
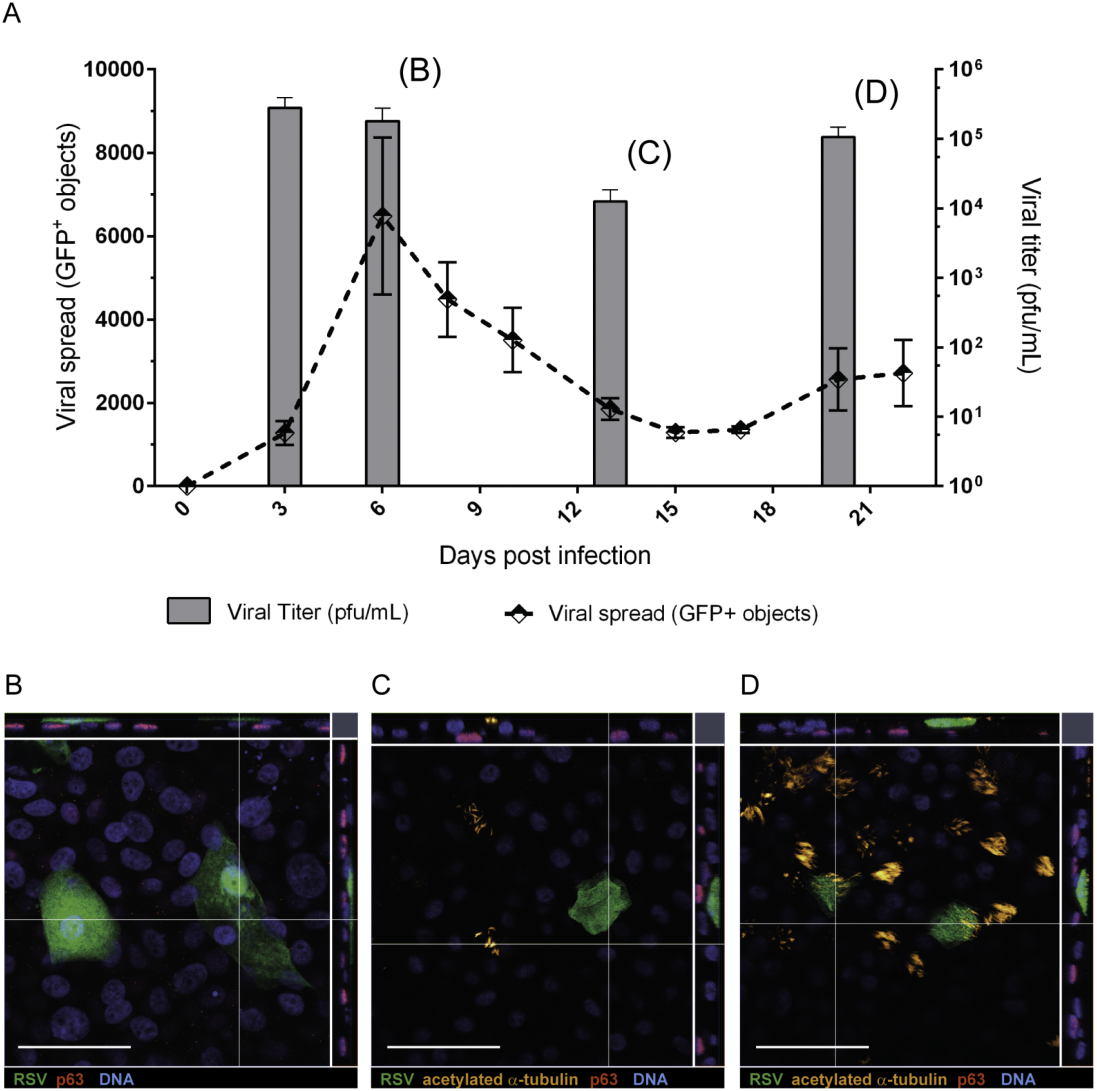
RSV infection of basal cells influences differentiation. The effects of RSV-A2-GFP infection of basal cells (100 pfu/insert, 3 h after seeding) were investigated over the duration of epithelial growth and differentiation. Infection of the epithelium was monitored by live cell imaging of GFP^+^ objects every 2–3 days (A; dotted line) whilst viral release was determined by plaque assay at day 3, 6, 13 and 21 (A; filled bars). Mean data ± SEM from 3 independent studies are shown. Representative planar and transverse views by confocal microscopy showing RSV-A2-GFP infected cells at day 6 (B), infected non-ciliated cells at day 13 (C) and infected ciliated cells at day 20 (D). GFP was used to visualize RSV-infection (green), acetylated α-tubulin stain for cilia (orange) and p63 for basal cells (red). Scale bars indicate 50 µm.

Having demonstrated that RSV could infect basal cells both before and after the formation of a well-differentiated epithelium, we asked whether infected basal cells could sustain viral replication and enable the virus to spread in the context of a polarized epithelium, as observed in the infant baboon [16] and pre-term lamb models [17]. To address this question we infected 100,000 basal cells before differentiation (3 h after seeding) with 100 pfu of RSV-A2-GFP, similarly to the experiment shown in Figure S1. Cells were cultured submerged in medium until day 7 and then exposed to ALI. Live cell images of the infected cultures were collected every 2–3 days over three weeks and GFP^+^ cells were quantified to monitor viral infection. This analysis showed that RSV spread rapidly in the basal cell population until day 6 (line graph in Figure 2A, B), a time point where the uninfected control cultures were still predominantly p63^+^ basal cells (data not shown). Viral titers in apical washes indicated that RSV was shed by the infected basal cells between days 3 and 6 post-seeding/infection (bar graph in Figure 2A). The numbers of GFP^+^ cells then declined to approximately 20% of the maximum (Figure 2A), although viral titers in the media remained elevated. At day 13, coinciding with the time where ciliated cells first became apparent (Figure 2C), the numbers of GFP^+^ cells began to increase, with infection of ciliated cells by day 20 (Figure 2D).

With evidence that an RSV infected basal cell could sustain viral replication, leading to spread of the infection throughout the course of epithelial formation and differentiation, we investigated how a range of viral doses could impact the epithelium. Basal cells infected with viral doses ranging from 1,000 pfu (MOI = 0.01) to 1 pfu (MOI = 0.00001) per insert formed a confluent monolayer with an intact barrier by 6 days post-seeding (equivalent to the behavior of uninfected cells), as judged by their ability to maintain a dry apical surface at ALI. Higher viral doses induced cell death and prevented the formation of a confluent cell monolayer so were not analyzed further (data not shown). Cells were examined at 21 days post infection to characterize their morphology and determine the extent of viral spread (Figure S2). At each of the viral doses examined, the infected cells were in an apical location (for example Figure 2D). However, the morphology of the infected cells and extent of viral spread was very different for the different viral doses (Figure S2A–D). Following infection with 1,000 pfu, numerous elongated, larger cells were apparent with evidence of syncytia (Figure S2A, E) and large, flat mono-nucleated cells (data not shown). The lower viral doses of 1 pfu and 10 pfu resulted in infected cells of a considerably smaller size (Figure S2C–D). The viral dose of 100 pfu generated RSV infected cells of small size as well as large flattened out cells (Figure S2B). Thus, even when inoculated at a low level, RSV was able to infect basal cells and spread in the basal cell population, resulting in widespread viral infection by the time of full differentiation.

### RSV infection of basal cells results in loss of ciliated and gain of secretory cells

**Figure 3:**
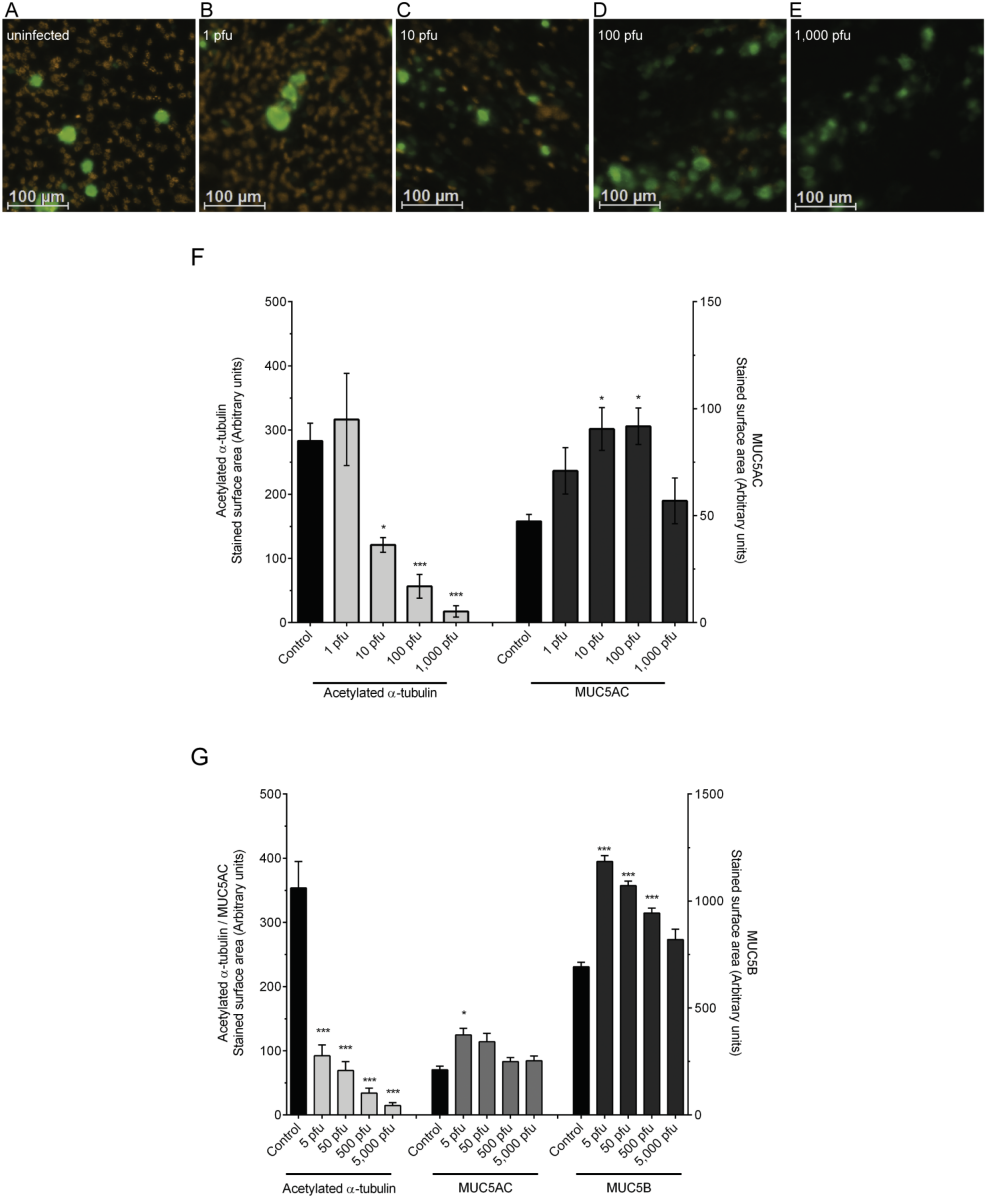
Epithelial composition following RSV infection. The effects of RSV-A2-GFP infection of basal cells on epithelial differentiation were assessed by quantitative immunofluorescence. Representative, images of HBEC cultures (21 days after seeding/infection) that were either uninfected (A) or infected with 1, 10, 100, or 1,000 pfu of RSV-A2-GFP (B–E, respectively). MUC5AC^+^ goblet cells and acetylated a-tubulin^+^ ciliated cells are pseudo-colored green and orange respectively. Staining for each of the cell types was quantified by image analysis (F). An alternative RSV strain, RSV-A2-MOT0972, was also assessed for effects on epithelial differentiation by quantitative immunofluoresence (G) as described above. For each study, mean data ± SEM from 6–9 inserts over three independent experiments are shown. Statistical significance was determined with a one-way ANOVA with post-hoc Dunnetts test compared to untreated control cells. * indicates p<0.05, **p<0.01 and ***p<0.001.

The data described above illustrate that in the presence of an ongoing RSV infection the airway epithelium was able to form a monolayer and differentiate. To determine whether RSV infection could influence the cellular composition of the epithelium, the cultures described above were assessed by quantitative immunofluorescence, using acetylated α-tubulin as a marker for ciliated cells and MUC5AC for secretory cells. At viral doses ≥10 pfu/Transwell (MOI: 0.0001), RSV infection resulted in a significant reduction in ciliated cells and an increase in MUC5AC^+^ secretory cells by day 21 (Figure 3). At lower doses of RSV (<10 pfu/Transwell) the cellular composition of the epithelium was similar to that of the uninfected control and the infected (GFP^+^) cells were exclusively ciliated cells (data not shown). This observation was verified in cells from three donors (Figure S3). In addition, to eliminate the possibility that the modified RSV-A2-GFP strain was not representative of wild-type RSV infection, we repeated the experiments using a clinical isolate designated RSV-A2-MOT092 (obtained from Dr. Edward Walsh, University of Rochester Medical School). For the clinical isolate we also had the possibility to stain for MUC5B in addition to MUC5AC and acetylated α-tubulin, as the GFP channel was not occupied by RSV-A2-GFP. For each experiment, one well was stained for RSV to verify viral infection (data not shown). RSV-A2-MOT0972 showed an identical epithelial phenotype with a loss of ciliated cells and an expansion of both MUC5AC^+^ and MUC5B^+^ secretory cells (Figure 3G). Together, these experiments illustrate that RSV infection of the progenitor basal cells induced a change in the phenotype of the resulting epithelium with an increased number of goblet cells and reduced number of ciliated cells.

### Type III IFNs contribute to the RSV-induced change in epithelial phenotype

**Figure 4:**
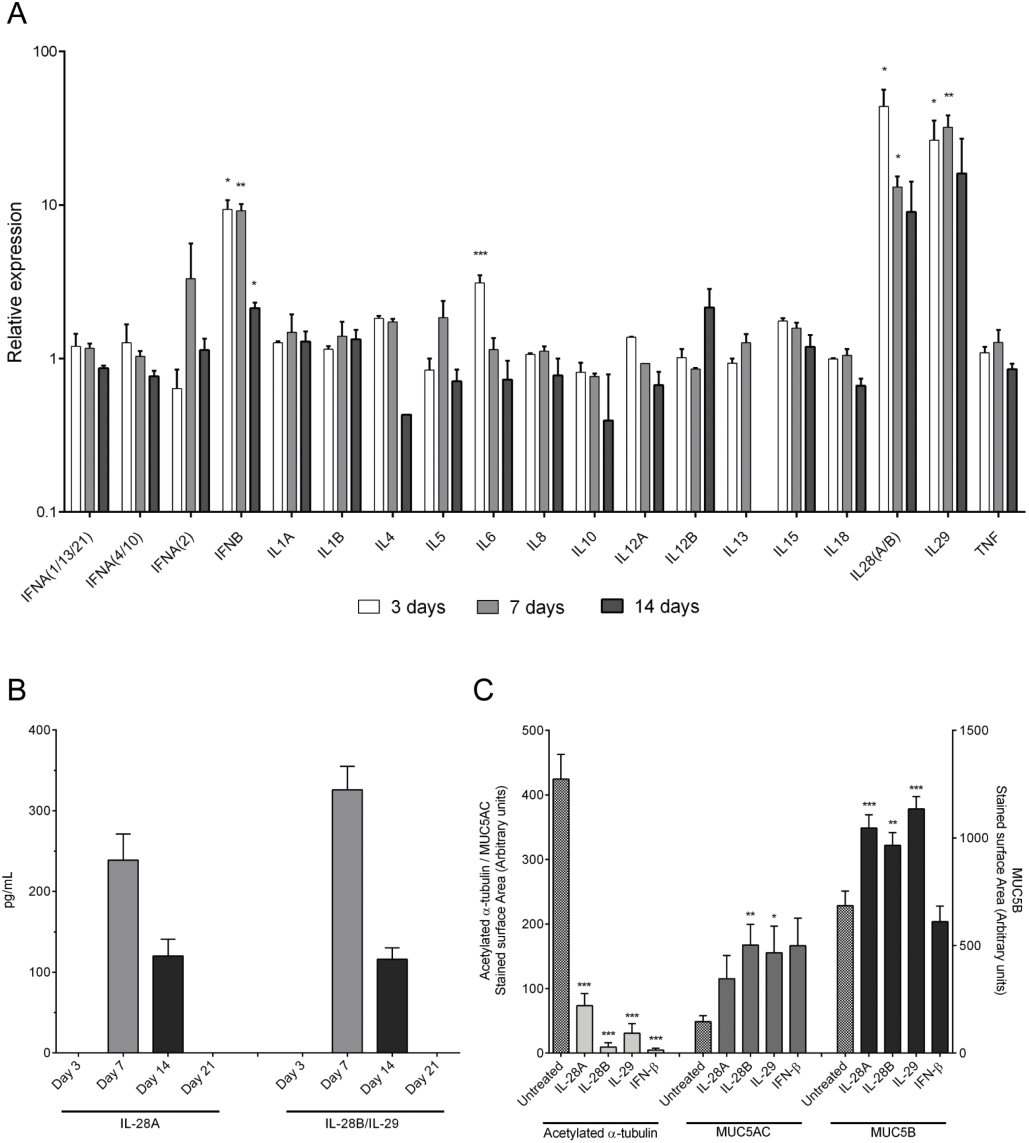
RSV infection induces an IFN-β and Type III interferon response in basal cells. The induction of an innate host response following RSV-A2-GFP infection of basal cells (100 pfu/insert, 3 h after seeding) was investigated over the duration of epithelial growth and differentiation. The expression of immune modulators was assessed at days 3, 7 and 14 after infection. qRT-PCR data are expressed relative to the time-matched, uninfected control (A). At day 3, 7, 14 and 21 media was collected and the levels of secreted IL-28A and IL-28B/IL-29 were determined by a sandwich ELISA (B). Quantitative immunofluorescence was used to assess the effects of IL-28A (30 ng/mL), IL-28B (30 ng/mL), IL-29 (30 ng/mL) or IFN-β (2.5 ng/mL) on epithelial differentiation (C). Mean data ± SEM from 9 inserts over 3 independent experiments are shown, except for panel B, in which 14 inserts over 5 independent experiments were analyzed. Statistical significance was determined with a one way ANOVA statistical test with post-hoc Dunnetts test compared to untreated control cells. *p<0.05, **p<0.01, and ***p<0.001.

RSV infection of the airway epithelium has been demonstrated to induce the release of a number of secreted factors that likely regulate the host immune response, including: IL-1α, IL-1β, IL-4, IL-5, IL-6, IL-8, IL-10, IL-12, IL-13, IL-15, IL-17A, IL-18 and TNF-α [7], [10], [34], [35], [36], [37], [38], [39], [40]. RSV has also been shown to induce a type I (IFN-α and IFN-β) and type III (IFN-λ) interferon responses in multiple cell lines [10], [12], [36], [39], [41], [42]. To assess the potential influence of the RSV-induced factors on the epithelial phenotype, we profiled their expression levels during differentiation. Basal cells were infected with 100 pfu of RSV-A2-GFP and expression levels of different soluble immune factors were determined by qRT-PCR at days 3, 7 and 14 days post-infection. Notably, RSV infection induced an early IL6, IFNβ and IFNλ (IL28A/B and IL29) response in the epithelium, which diminished over time (Figure 4A; note that due to the high similarity between IL28A and IL28B, our probes did not differentiate between the two). IFNγ and IL17A mRNAs were below the level of detection at each time point examined (data not shown) and there was no evidence for significant regulation of the other gene transcripts studied (Figure 4A). The RSV-induced changes in IFNλ mRNA levels correlated with a significant increase in levels of both IL-28A and IL-28B/IL-29 protein, which were detectable from day 7. The levels of these interferons declined over time and were undetectable at day 21 (Figure 4B). It was not possible to determine whether IL-28B was induced, as that interferon was grouped with IL-28A in the gene expression analysis and with IL-29 in the ELISA. The time for maximum concentrations of IL-28A, IL-28B and IL-29 correlated with the peak release of viral particles (bar graph in Figure 2A), consistent with the predicted anti-viral response of the epithelium. Secreted IFN-β was below the level of detection at each time point examined (data not shown). We did not perform any further studies on IL6 as IL6 was not significantly up-regulated at 7 days post infection when the cells were brought to ALI to differentiate and was therefore unlikely to affect epithelial composition.

We next asked whether treatment with the RSV-induced cytokines could phenocopy the effect of RSV infection on the epithelial composition of air-liquid interface cultures. Concentrations of the different interferons were selected based on an initial pilot study that covered the range of 0.3 to 30 ng/mL for each. Based on this study, IL-28A, IL-28B and IL-29 were added at a concentration of 30 ng/mL with IFN-β being tested at 2.5 ng/mL as higher concentrations of IFN-β did not allow for a confluent cell layer to be formed. Cells were treated from days 0–21 in culture with fresh aliquots of mediator being added on every feeding. Treatment with IL-28A, IL-28B, IL-29 or IFN-β resulted in an epithelial phenotype similar to that formed following RSV infection, with a significant loss in ciliated cells and expansion of MUC5AC and MUC5B secretory cells (Figure 4C). The epithelial remodeling caused by IL-28A, IL-28B, IL-29 and IFN-β indicated that the loss of ciliated cells observed during RSV infection could be driven by a type I and/or type III IFN response.

**Figure 5:**
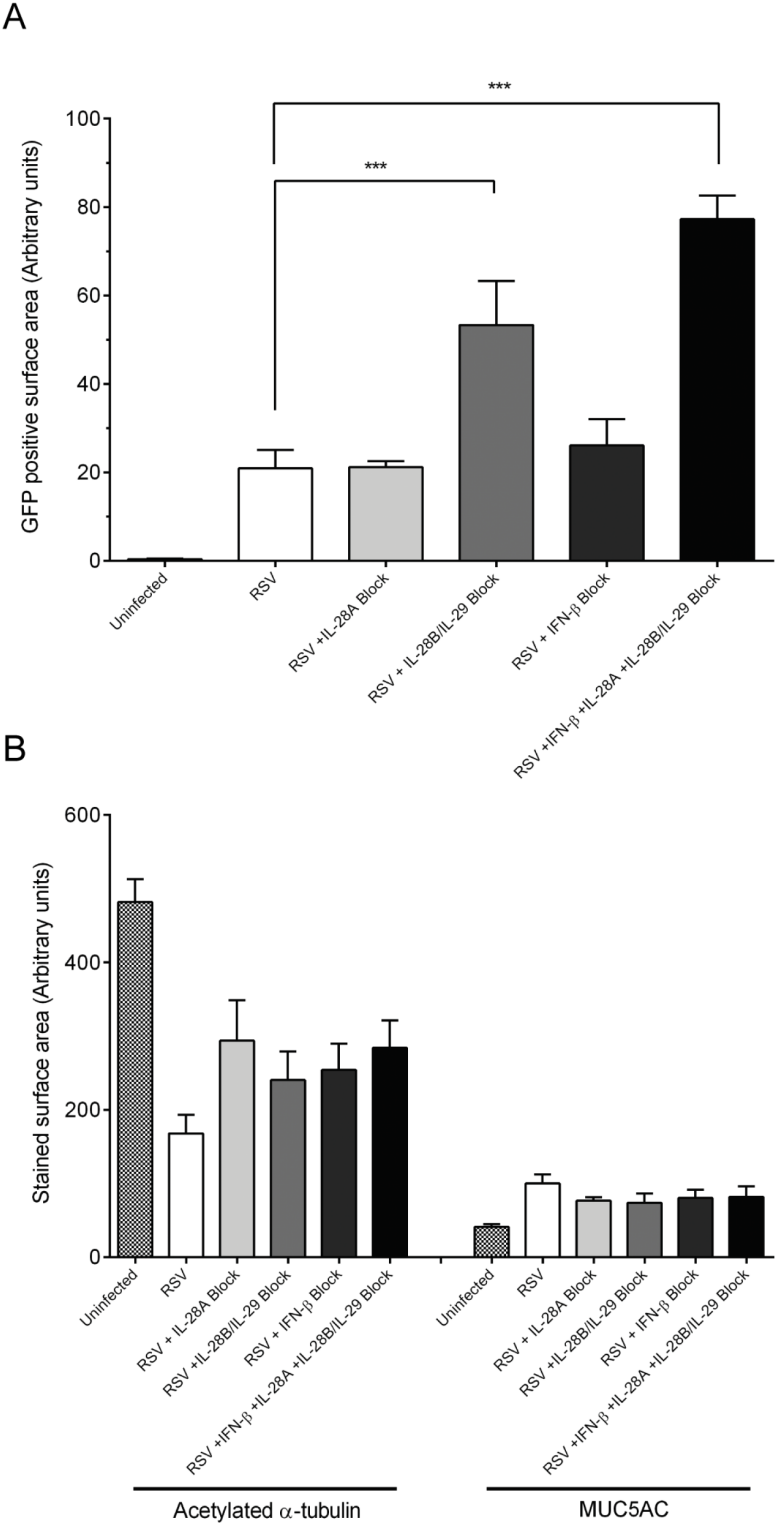
Neutralization of the IFN response during RSV-A2-GFP infection. The effects of neutralization of IL-28A, IL-28B/IL-29 and IFN-β following RSV infection were assessed. GFP^+^ surface area was used to assess the degree of RSV infection (A) and quantitative immunofluorescence was used to determine the balance of acetylated α-tubulin^+^ and MUC5AC^+^ cells. Mean data ± SEM are shown from 10–14 inserts over 4–6 independent experiments, respectively. Statistical significance was determined with a one way ANOVA statistical test with post-hoc Dunnetts test compared to untreated control cells, ***p<0.001.

Finally, to address whether the RSV-induced production of IFN-β or the type III interferons are responsible for the observed change in epithelium, we neutralized the interferon response using antibodies directed against each of the induced ligands IL-28A, IL-28B, IL-29 and IFN-β, both as combination treatment and individually. All of the antibodies used for this experiment were verified to have neutralizing activity (Figure S4). One hundred pfu of RSV-A2-GFP was added to each well of HBECs 3 h post seeding and interferon neutralizing antibodies or isotype controls were included in the medium from day 0 to 21. The antibodies were replaced at each media exchange and at day 21 the cells were fixed and analyzed by quantitative immunofluorescence. Neutralization of the complete IFN response using a combination of all three antibodies caused a 3 to 4-fold increase in RSV spread (Figure 5A), demonstrating the importance of the innate immune response in limiting viral replication. Neutralization of IL-28B/IL-29 alone gave a similar increase in viral spread as combination treatment (Figure 5A), consistent with a key anti-viral role in limiting replication and spread. Next we assessed epithelial composition during interferon neutralization by quantitative immunofluorescence. We reasoned that if interferons had no effect in the epithelial composition, neutralization of the IFN ligands would result in reduced numbers of ciliated cells because of the increased degree of RSV infection under these conditions. However, neutralization of either all ligands together, or independently, resulted in similar numbers of ciliated cells compared to cells treated with an isotype control (Figure 5B). This result indicates that the interferons released as a consequence of RSV infection are at least in part responsible for causing the reduction in numbers of ciliated cells. The neutralization of the interferon response likewise had no effect on secretory cell numbers despite the increase in the number of infected cells. Taken together, these results show that RSV infection elicited an innate immune response of IFN-β and type III interferons at the transcriptional level together with detectable secretion of the type III interferons. Neutralization of the type III interferon response enhanced the viral infection but also at least partially protected the epithelium from loss of ciliated or gain of secretory cells.

## Discussion

In this study we present data demonstrating that: 1) following epithelial injury, RSV could infect p63^+^ airway basal cells, and 2) RSV infection of proliferating basal cells profoundly influenced the subsequent cellular composition of the epithelium. The airway basal cell is the key progenitor of the human conducting airway epithelium and the regulation of epithelial turnover, repair and subsequent differentiation are essential to maintain barrier function and protect the lung from inhaled pathogens and allergens. The present study provides the first evidence that RSV infection of primary human airway basal cells can divert epithelial differentiation towards a phenotype that has increased numbers of goblet cells and reduced numbers of ciliated cells. This phenotype was replicated using two different viral strains (Figure 3) in three different donors (Figure S4). These *in vitro* findings share a striking resemblance to those frequently observed in the human host with RSV infection, where patients have fewer ciliated cells and an increased number of secretory cells [43], [44].

The relevance of the airway basal cell as an RSV-sensitive cell -type is somewhat controversial. Several studies have demonstrated that the ciliated cell is susceptible to infection by RSV *in vitro* [11], [12], [15] as well as in humans *in vivo* [24], [26]. Indeed, the well-differentiated ALI HBEC system we have used also demonstrates a clear infection of the ciliated cell when RSV is added to the apical surface in the normal, steady-state system (Figure 1B). However, in a number of respiratory diseases the airway epithelium can be damaged and barrier function impaired. Potentially, this could expose the basal cell to pathogens and induce basal cell proliferation. Indeed, the Gern group recently demonstrated that impairing the barrier properties of the human airway epithelium in vitro, enabled infection of the otherwise inaccessible basal cell with rhinovirus [33]. In the case of RSV, there is some evidence to suggest that basal cells might become infected under certain conditions. Pathology studies have described RSV infected cells that do not have cilia, although their identity was not investigated further [24]. In addition, there are reports based on two animal models, infant baboons and pre-term lambs, which describe infection of airway basal cells [16], [17] although the identity of the infected cell-type was not confirmed using cell-specific markers. Based on these findings, we considered that a re-evaluation of basal cell tropism for RSV and potential consequences of infection should be undertaken.

The overt RSV infection of p63^+^ basal cells that we have observed in ALI cultures of HBEC is in contrast to the report of Zhang et al [11]. However, it should be noted that in that study, even though it was stated that no basal cells were infected after a scratch injury, infected cells were observed in and around the wound area, although their identity was not described [11]. Our data clearly show that irrespective of the method of epithelial injury, mechanical or chemical, RSV infected p63^+^ basal cells were observed within hours of a brief exposure to virus. It is difficult to reconcile the differences in observations between the published scratch wound model and our own other than that we used cells at 2–4 weeks following the establishment of ALI as compared with 4–6 weeks. In both cases, cultures were well differentiated (evidenced by the presence of ciliated cells) although to our knowledge, any putative differences in basal cell populations at these times have not been described. Of note, the magnitude of p63^+^ cell infection in the present study was extensive and far from being isolated to an occasional, rare cell type (Figure 1C–I) further supporting the concept that exposure of basal cells to RSV in a susceptible airway could result in significant infection.

Having established that basal cells within the mature ALI cultures could become infected with RSV following mechanical or chemical damage, we demonstrated that freshly seeded basal cells were also susceptible to infection (Figure 2, S1). Freshly seeded basal cells are highly proliferative as the cells attempt to ‘repair’ the epithelium. As such, a distinction should be made between the behavior of these dividing cells and the relatively quiescent basal cell in the healthy, fully developed epithelium. The lack of basal cell infection observed by Zhang et al [11] may relate to this difference in proliferative status of the cells. However, this would still not account for the differences in susceptibility to RSV infection following scratch injury between this work and the present study.

Using live cell imaging to follow the infected epithelium over time, it was clear that the initial infection of the basal cells was sustained and resulted in viral propagation that persisted until at least 21 days in culture (Figure 2, S2). As described above, even very low viral doses were capable of initiating a sustained infection that continued to generate infectious viral particles throughout the entire period of epithelial growth and differentiation. Between days 0–6 and then again from day 17 onwards, the numbers of infected cells in the epithelium increased. However, despite sustained infectious virus release, the numbers of infected cells declined from days 6–15. The failure to continue to infect the epithelium during days 6–15 is consistent with the work of Zhang et al [11], who showed a loss of susceptibility to RSV infection over this same period of culture. This may have been due to the concurrent induction of a protective interferon response (Figure 5B, 6A) that in some way limited spread and/or susceptibility of the epithelial cells to RSV. Accessibility of RSV to the basal cell may also account for the loss of susceptibility to infection at this time as confocal imaging of HBEC revealed a multilayered epithelium by day 6 in culture, with the p63^+^ basal cell being covered by a p63^−^ cell type (Figure 2B). Of note, the increase in infected cells from day 17 would coincide with the appearance of ciliated cells in the epithelium from day 13.

**Figure 6:**
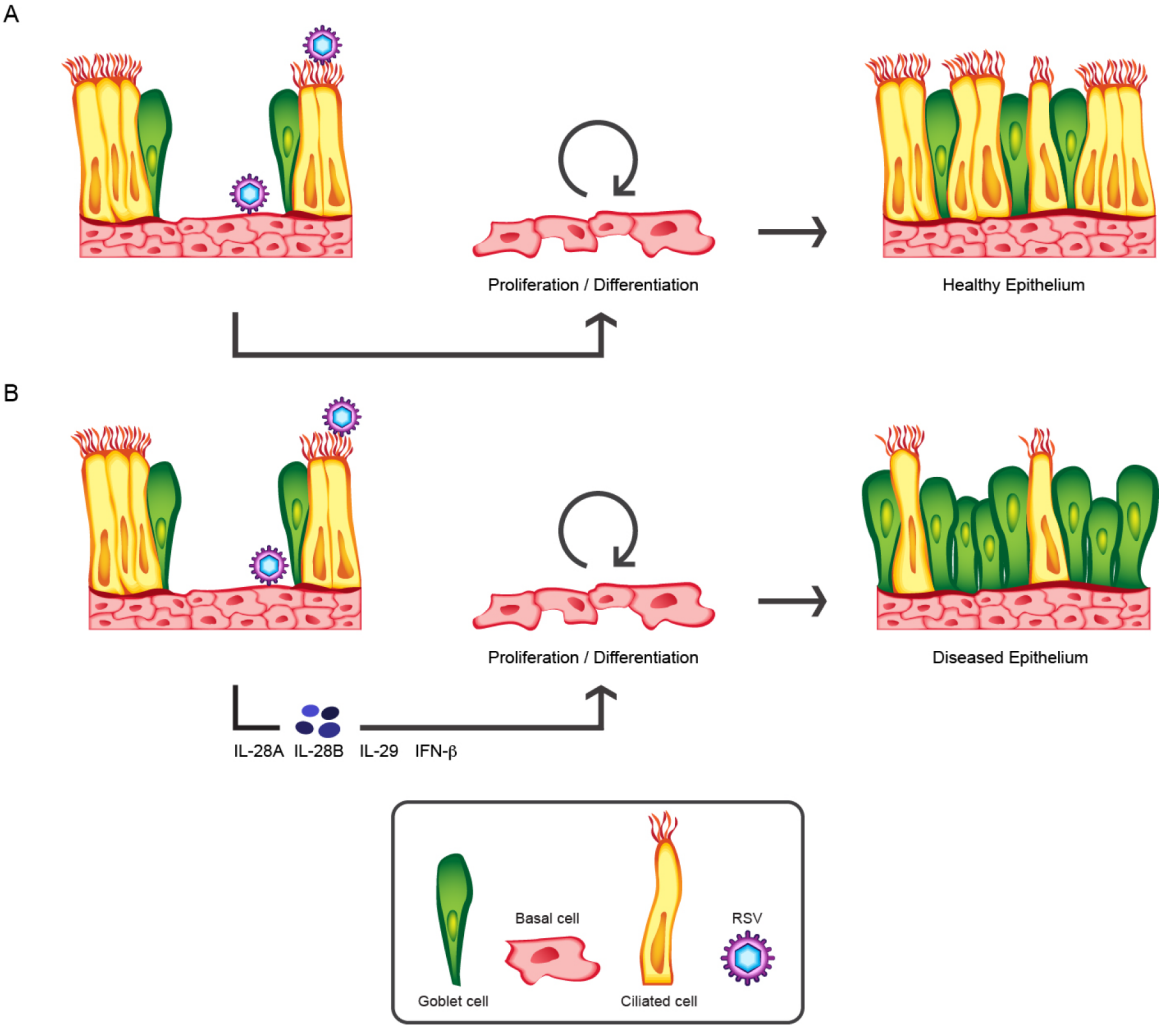
Proposed model for the impact of RSV infection of the airway basal cell. The multi-potent airway basal cell plays a central role in epithelial repair following injury, resulting in an epithelium composed of both ciliated and goblet cells (A). In patients with an impaired epithelial barrier, resulting from either the natural history of an RSV infection (epithelial sloughing) or because of a pre-existing respiratory disease, the airway basal cell can now become exposed to inhaled RSV particles (B). Infection of the basal cells by RSV results in the release of IL-28A, IL-28B, IL-29 and IFN-β that influences basal cell differentiation towards a hypersecretory phenotype i.e. gain of goblet cells and loss of ciliated cells that has the potential to contribute towards a disease exacerbation.

Interestingly, the initial viral dose did not necessarily relate to the final extent of infection. RSV doses of 1, 10 and 100 pfu showed an inverse correlation with the degree of infection by day 21 (Figure S2). A possible explanation for this is that low viral doses enabled a relatively normal epithelium to be formed with a relatively high proportion of ciliated cells for RSV to infect at later time points. It is remarkable that the degree of RSV infection appeared to reach a plateau at 20 days, even with a high number of uninfected ciliated cells present. This could imply that ciliated cells are infected relatively inefficiently by RSV (Figure 2) or that sub-populations exist with different viral susceptibilities. Support for this comes from the fact that even inoculation of well-differentiated HBEC cultures containing numerous ciliated cells, with high doses of RSV, or after an extended period of time, resulted in infection of only a fraction of this cell population (Figure 1A–B and Figure 2). Together, our data suggest that exposed basal cells were readily infected by RSV even when very low viral doses were used suggesting that RSV infection of the basal cells could be an underappreciated feature of RSV pathogenesis.

The data presented in Figure 3 provides strong evidence that RSV infection of airway basal cells resulted in a well-differentiated epithelium, but one that lacked ciliated cells and had increased numbers of secretory cells leading to a goblet cell hyperplasia and a hypersecretory phenotype [45]. The observed attenuation of p63 expression in infected basal cells may be consistent with an RSV-dependent switch in basal cell fate. A loss of p63 expression is essential to the commitment of undifferentiated dividing cells to undergo maturation [46]. It is also possible that RSV infection of emerging ciliated cells early in the course of differentiation led to a depletion in this cell type. In the context of human infection, such a change in epithelial phenotype may be a beneficial host response to limit viral spread by both limiting the numbers of ciliated cells and creating a localized mucus gel. Conversely, infection of the epithelial progenitor could drive an excessive switch in phenotype that may contribute to disease morbidity. At the higher viral doses, RSV resulted in some enlarged infected cells in the apical region of the epithelium, that was most pronounced at 1,000 pfu (Figure S2). These enlarged cells were negative for ciliated or secretory cell markers, were not apoptotic, but were often positive for cytokeratins 5 and/or 8 (data not shown). Similar enlarged multinucleated cells have been observed previously both in HBEC ALI cultures infected with RSV [15] and in post mortem biopsies from young children who succumbed to respiratory disease [24].

In view of the influence that soluble mediators such as IL-1β, IL-9 and IL-13 [47], [48], [49] can impart on airway epithelial formation, we considered the possibility that soluble factors induced by RSV infection might be modifying the epithelial phenotype. A number of secreted factors have been demonstrated to be released following infection with RSV, including: IL-1α, IL-1β, IL-4, IL-5, IL-6, IL-8, IL-10, IL-12, IL-13, IL-15, IL-17A, IL-18, TNF-α, IFN-α, IFN-β and IFN-λ [7], [10], [34], [35], [36], [37], [38], [39], [40]. Of these, only IL-6, IFN-β and the Type III interferons were expressed by the HBEC during the course of RSV infection (Figure 5A). It was surprising that IL-13, a widely recognized driver of goblet cell formation in the airways [49], was not induced by RSV infection in the present model system. Published studies have shown IL-13 to be induced following RSV infection [14], [50], [51], [52]. The lack of effect in the present study may relate to the timing of infection (early basal cell infection versus infection of a mature epithelium) and/or the 5,000-fold lower viral dose we used. Of note, differences in innate immune responses between basal cells and a well-differentiated epithelium after RSV infection have been previously reported [13].

The RSV-induced expression of IFN-β and the type III interferons at the transcriptional level (Figure 4A) translated through to the secretion of detectable levels of IL-28A, IL-28B and IL-29 (Figure 4B). Even with the delay between mRNA and protein levels of IL-28A, IL-28B and IL-29 the concentrations peaked at a time point that correlated very well with high viral release, consistent with interferons playing an anti-viral role in RSV pathology. The induction of IFN-α/β by RSV has previously been reported in ALI HBEC [12], but a type III interferon response has so far only been described in nasal epithelial cells [41]. We were unable to detect significant levels of secreted IFN-β, consistent with the findings by Villenave et al. [15]. Neutralization of the endogenous IL-28B and IL-29 that was produced following RSV infection significantly increased the numbers of infected cells, supporting the anti-viral role for these mediators in the human airway epithelium. IL-28A and IFN-β neutralization did not affect the magnitude of the viral infection suggesting that these mediators have a less relevant, or even absent, role in the anti-viral response of the epithelium to RSV. That IL-28A neutralizing antibodies had no effect on viral spread was surprising as IL-28A is reported to share a common receptor and signaling pathway with IL-28B and IL-29 [53]. Pilot studies using A549 cells did confirm the neutralizing capacity of the anti-IL-28 antibody and selected dose (Figure S4), however it is possible that this failed to translate through to efficacy in a different cell type and in a study with an extended duration of exposure (24 h vs. 21 days). Alternatively, there may be a divergence of the signaling pathways between IL-28A and IL-28B/IL-29 in the HBEC cultures. With the interferon response maintained throughout the period of epithelial differentiation we addressed whether these mediators could influence the differentiation process. Exogenous addition of the IFN-β and the type III interferons had the capability to phenocopy the effect of RSV infection on the epithelial composition (Figure 4C). Remarkably, despite the significantly enhanced viral infection of the IL-28B/IL-29 neutralized epithelium, the numbers of ciliated and secretory cells were unchanged from the IL-28B/IL-29 intact control. Based on the earlier dose-response data (Figure 3), it would have been predicted that the increased infection should have further reduced ciliated cell number and boost secretory cells. These data are therefore consistent with RSV-induced levels of endogenous IL-28B and IL-29 contributing towards both the loss of ciliated and gain of secretory cells.

Together, these data highlight that RSV infection of basal cells could have a significant, long-term impact on the biology of the airway epithelium (Figure 6). According to this model infection of the basal cells enables rapid and efficient RSV replication and spread of the infection. The infected epithelium mounts an early interferon response that plays a central role in the anti-viral response of the epithelium. However, in addition to the anti-viral effect of the endogenous production of IL-28B/IL-29, these mediatorscan also repress ciliated cell formation and increase secretory cell numbers. The impact is to push epithelial differentiation away from a ‘normal’ ciliated phenotype (Figure 6A) and towards a ‘disease-like’ pro-secretory function (Figure 6B). If this were to happen in infection of an individual, this change in epithelial phenotype may contribute to the excessive mucus production and occlusion of small airways that can occur following RSV infection. The balance between secretory and ciliated cells in the lung is critical to maintain a normal lung function. A degree of epithelial plasticity is beneficial to the healthy lung, allowing it to respond to injury by increasing the production of the protective mucus gel and in the case of RSV, reducing the number of susceptible ciliated cells. In diseases such as cystic fibrosis, COPD and asthma however, where epithelial barrier function can be compromised and the balance is already skewed towards the goblet cell, RSV infection of the basal cell might be predicted to further amplify that phenotype. Excessive mucus together with a reduced ciliated cell population can lead to direct airway occlusion, mucostasis and an increased risk of further infection or even bacterial colonization. As such, we would propose that infection of the basal cell by RSV may be a significant contributor to exacerbations of respiratory diseases and should be further investigated in human pathology. Strategies to regulate either the initial infection or response of airway basal cells may provide novel approaches for generating broad acting therapeutics under healthy or exacerbated conditions important for viral lung disease.

## Methods

### Cells and viruses

Human bronchial epithelial cells (HBEC) were purchased from Lonza and expanded in Bronchial Epithelial cell Growth Medium (BEGM) supplemented with the recommended additives (Singlequots, Lonza) until 80% confluent (P0 cells). Once confluent the cells were aliquoted and frozen down as ‘P1’ cells. For each experiment P1 cells were expanded to 80% confluence and seeded onto a 12-well Transwell permeable support (0.4 µM, Corning, NY) in BEGM media diluted 1∶1 in DMEM (Lonza, Invitrogen) and supplemented with BEGM singlequots devoid of triiodothyronine (T3) and retinoic acid (differentiation media). All trans-retinoic acid (Sigma, 50 nM in ethanol) was added back fresh for each media exchange. Cells were infected by either RSV-A2-GFP [31], [32], or a clinical isolate designated RSV-A2-MOT0972 (Obtained from Edward Walsh, University of Rochester Medical School) at various multiplicity of infection (MOI), as indicated in the text. Six days post-seeding the media was removed from the apical side and cells were cultured at ALI for an additional 14 days. Media was replaced three times a week with 1 mL on the basal side and for week one, 400 µL was added on the apical side.

### RSV quantification

RSV-A2-GFP was grown in HEp-2 cells and purified by centrifugation through sucrose. Briefly, cells were infected with RSV at an MOI of 0.05. Ninety-six hours post-infection the cells were scraped into the media, which was collected and vortexed to release cell-associated viral particles. Cell debris was pelleted by centrifugation and the supernatant was loaded onto a 20% sucrose cushion in PBS and purified by centrifugation at 11,500 g. Pelleted virus was re-suspended in PBS containing 25% sucrose and flash frozen in liquid nitrogen. Aliquots of the virus were stored at −80°C. To quantify released virus from infected HBEC, 500 µL of warm DMEM was added to the apical side for 60 min and then collected. Aliquots were flash frozen in liquid nitrogen. All viral titers were determined by a conventional plaque assay. Ten-fold dilutions of cell supernatant were used to infect HEp-2 cells before overlaying with OptiMEM (Invitrogen) containing: 2% FCS (Invitrogen), 0.8% Methylcellulose (Sigma) and 1x Penicillin/Streptomycin (Invitrogen). Five days post-infection the cells were fixed with 80% cold methanol for 2 h, rinsed with ddH_2_O and blocked with 5% dried milk in PBS. RSV infected cells were stained by RSV-F antibody (Serotec MCA490) followed by horseradish peroxidase conjugated secondary antibody (Abcam). Plaques were visualized by 4CN peroxidase substrate (KPL).

### Epithelial denuding

Well-differentiated HBECs (at 21 days after seeding onto Transwell inserts) were rinsed three times in warm PBS (Invitrogen) to remove mucus. Once cleaned, warm S-MEM media (Invitrogen) was added for 20 min at 37°C (1 mL on the basal side and 500 µL on the apical side). Once separated the suprabasal layer was removed from the basal cell layer by vigorously pipetting up and down. The basal cell layer was washed several times using warm PBS until a single layer of cells could be observed. At this point cells were infected using 100,000 pfu of RSV-A2-GFP.

### Scratch injury

100,000 cells were seeded onto a Transwell insert and grown to form a pseudostratified epithelium over 21 days as previously described [11]. Once fully differentiated the cells were washed once from the apical side using warm PBS followed by addition of 500 µL PBS. A GUM stimulator (generic dental tool) was used to carefully scratch several thin scratches that intersected each other. Once scratched 250,000 PFU of RSV-A2-GFP was added in 250 µL of warm HBEC growth media for 30 min at 37°C. After 30 min the apical media was removed, cells incubated at 37°C for 20 h followed by fixation using 4% paraformaldehyde.

### Flow cytometry

100,000 cells were seeded onto a Transwell insert infected 3 h later with RSV-A2-GFP at an MOI of 0.1. Uninfected control cells were incubated with the respective volume of PBS. After 20 h cells where harvested and fixed by 2% paraformaldehyde for 10 min at RT, centrifuged for 5 min at 1,500 rcf and washed once in PBS. Fixed cells were permabilized by PBS+0.5% Triton X-100 for 15 min at RT, followed by washing once in PBS. Fixed and permabilized cells were stained for p63 (Abcam ab124762) and ITGA6 (Millipore MAB1378) at a dilution of 1∶200 for 45 min, washed once, and incubated with an Alexaflour 568 and Alexaflour 647 conjugated secondary antibody (Invitrogen) at 1∶1000 for 30 min. Flow cytometry was performed in PBS using a Fortessa cytometer.

### Immunohistochemistry, live cell imaging and cell imaging analysis

Cells were fixed by 4% paraformaldehyde for 3 h at 4°C and blocked using 10% horse serum in immunofluorescence buffer containing: 130 mM NaCl, 13 mM Na_2_HPO_4_, 3.5 mM NaH_2_PO_4_, 0.2% Triton X-100 and 0.05% Tween-20 at pH 7.5. All imaging was performed using a Carl Zeiss spinning disk confocal microscope using antibodies against: acetylated α-tubulin (Sigma T6793) to stain ciliated cells, MUC5B (Santa Cruz sc-20119) and MUC5AC (Thermo Scientific MS145P) for secretory cells and p63 (Abcam ab124762) for basal cells. For staining RSV-A2-MOT0972 infected cells a goat anti-RSV antibody was used (Pierce PA1–7240). All Alexa-conjugated secondary antibodies were obtained from Invitrogen. To quantify epithelial composition quantitative immunofluorescence was performed by collecting 130 overlapping images at 10x magnification, for each 1 cm^2^ Transwell insert, in Axiovision 4.8 (Carl Zeiss). This enabled almost the entire surface area of the insert to be captured and used for quantification removing any potential for operator bias. RSV-A2-GFP fluorescence was imaged using the same acquisition protocol. Converted images were analyzed by ImageJ [54] by measuring stained surface area over a pre-defined threshold, using the analyze particles routine. Data was normalized to uninfected control. For confocal microscopy, sections of the inserts were punched out using a 4 mm biopsy punch and mounted on a cover slide using ProLong Gold antifade reagent with DAPI (Invitrogen).

### Quantitative PCR

Total RNA was harvested and isolated by using the RNeasy kit from Qiagen, according to the manufacturer’s instructions (Qiagen). cDNA was generated by using the TaqMan reverse transcription reagents (Applied Biosystems) according to the manufacturer’s instructions. All FAM probes were purchased from ABI (Invitrogen) as 20x Master mixes and designed for maximum gene coverage (Table S1). Gene expression reactions were performed with TaqMan 2x PCR Master Mix (Applied Biosystems) in a ViiA7 Real time PCR cycler (Applied Biosystems). All the data is expressed as expression relative to the untreated control.

### ELISA

Basal supernatants were collected at day 3, 7, 14 and 21 post-infection. Uninfected samples were collected at the same time points. Collected supernatant was aliquoted and frozen until assayed for IL-28A or IL-28B/IL-29 production using a sandwich ELISA (R & D Systems). ELISA was performed according to the manufacturer’s instructions. IFN content was compared to a 7-point 2-fold dilution standard curve from 8,000 pg/mL or 4,000 pg/mL.

### IFN treatment

Soluble interferons were used at 30 ng/mL for IL-28A (R&D Systems), IL-28B (R&D Systems) and IL-29 (Gibco). For IFN-β (pbl Interferon Source) 2.5 ng/mL was used. Mediator was present in both apical and basal media for week one, and in the basal media once brought to ALI at day 6. Media was replaced three times a week and fresh mediator added. The experiment was terminated after 21 days by fixation using in 4% paraformaldehyde. Fixed cells were stained for epithelial composition using antibodies specific to acetylated α-tubulin, MUC5AC and MUC5B.

### Blocking of IFN response

We used antibodies against IL-28A (R&D Systems at 1 µg/mL), IL-28B/IL-29 (R&D Systems 12.5 µg/mL) and IFN-β (R&D Systems at 2 µg/mL) to neutralize the induced IFN response. Concentrations used had been verified to neutralize an induced IFN response in A549 cells to at least 50% (Figure S4). All treatments were performed as combination treatment or individual with matched isotype control. One week after seeding the media was removed from the apical side and cells were allowed to differentiate. Mediator was present in both apical and basal media for week one. The antibody treatment continued for the full 21 days. At day 21 cells were fixed by 4% paraformaldehyde and stained for epithelial composition using antibodies specific to MUC5AC and acetylated α-tubulin.

### Analysis of results

Data are presented as mean ± SEM unless otherwise stated. All statistical analyses were performed in GraphPad 6 (GraphPad software Inc., San Diego, CA) using analysis of variance (ANOVA) followed by a Dunnett’s multiple comparison test. Significance was assumed when p<0.05.

## Supporting Information

Figure S1 **Flow cytometry analysis of RSV-A2-GFP infected basal cells.** RSV infection of HBEC cells at 3 h post seeding allowed for an extensive infection of basal cells. At 45 h post infection cells were harvested, fixed and stained for the basal cell markers p63 and ITGA6. Close to 100% of the cells on the inserts were basal cells as indicated by positive p63 and ITGA6 stain (A–B). In addition, infected cells showed a significant reduction in p63 levels but not ITGA6 (C–D). Presented are representative flow cytometry data from three independent donors.
(TIF)

Figure S2 **RSV-A2-GFP infection 21 days post infection.** The effect of RSV-A2-GFP infection of basal cells (1–1,000 pfu/Transwell, D to A respectively, 3 h after seeding) was investigated over the duration of epithelial growth and differentiation by fluorescence imaging. Lower viral doses (C–D) exclusively generated infected cells with a condensed-morphology that gradually disappeared as the viral dose increased (A–D). The highest viral dose studied (1,000 pfu) resulted in the formation of an epithelium with large patches of infection (A, GFP^+^ cells) that were confirmed as syncytia by confocal imaging (E). Representative images from three independent experiments are shown, RSV infected cells are colored green.
(TIF)

Figure S3 **RSV induced epithelium phenotype was independent of donor.** HBEC cells from three different donors were investigated to rule out any for donor variability in the loss of cilia phenotype. Cells were infected at a Transwell insert 3 h after seeding using a range of RSV-A2-GFP from 1–1000 pfu/Transwell. After 21 days in culture the cells were stained for cilia using acetylated α-tubulin. Cells from all donors were cultured and imaged in parallel and three inserts from each donor was examined. Data is presented as the average ± SD, from 2 independent experiments and a total of 4–6 inserts per viral dose.
(TIF)

Figure S4 **Validation of neutralizing activity of anti-interferon antibodies.** Antibodies intended to neutralize IL-28A, IL-28B, IL-29 and IFN-β were all verified to neutralize a stimulated response in A549 cells. A549 cells were seeded into a 12 well plate (1.2×10^5^ cells/well) and stimulated for 2 h using 10 ng/mL of IL-28A (R & D Systems), IL-28B (R & D Systems) or IFN-β (pbl bioscience). The increasing concentrations of neutralizing antibodies added were based on the manufacturer’s neutralization data. After 24 h of treatment total RNA was collected using Buffer AVL from the RNAeasy kit (Qiagen) and RNA purified according to the manufacturer’s instructions. qRT-PCR was performed using 40 ng of total cDNA analyzing ISG15 for IL-28A/IL-28B/IL-29 stimulation (A–B) and CXCL10 for IFN-β stimulation (C). The concentration of antibody that resulted in a >50% pathway inhibition was used in the experiments presented in Figure 6.
(TIF)

Table S1 **qRT-PCR primer used and accession number.** All primers and probes where ordered from Invitrogen with the ordering number listed below.
(XLSX)
