# Supplementary figures and images for: Respiratory Syncytial Virus Can Infect Basal Cells and Alter Human Airway Epithelial Differentiation
Source: PLoS One. 2014 Jul 17;9(7):e102368. doi: 10.1371/journal.pone.0102368 (PMC4102526; doi:10.1371/journal.pone.0102368)

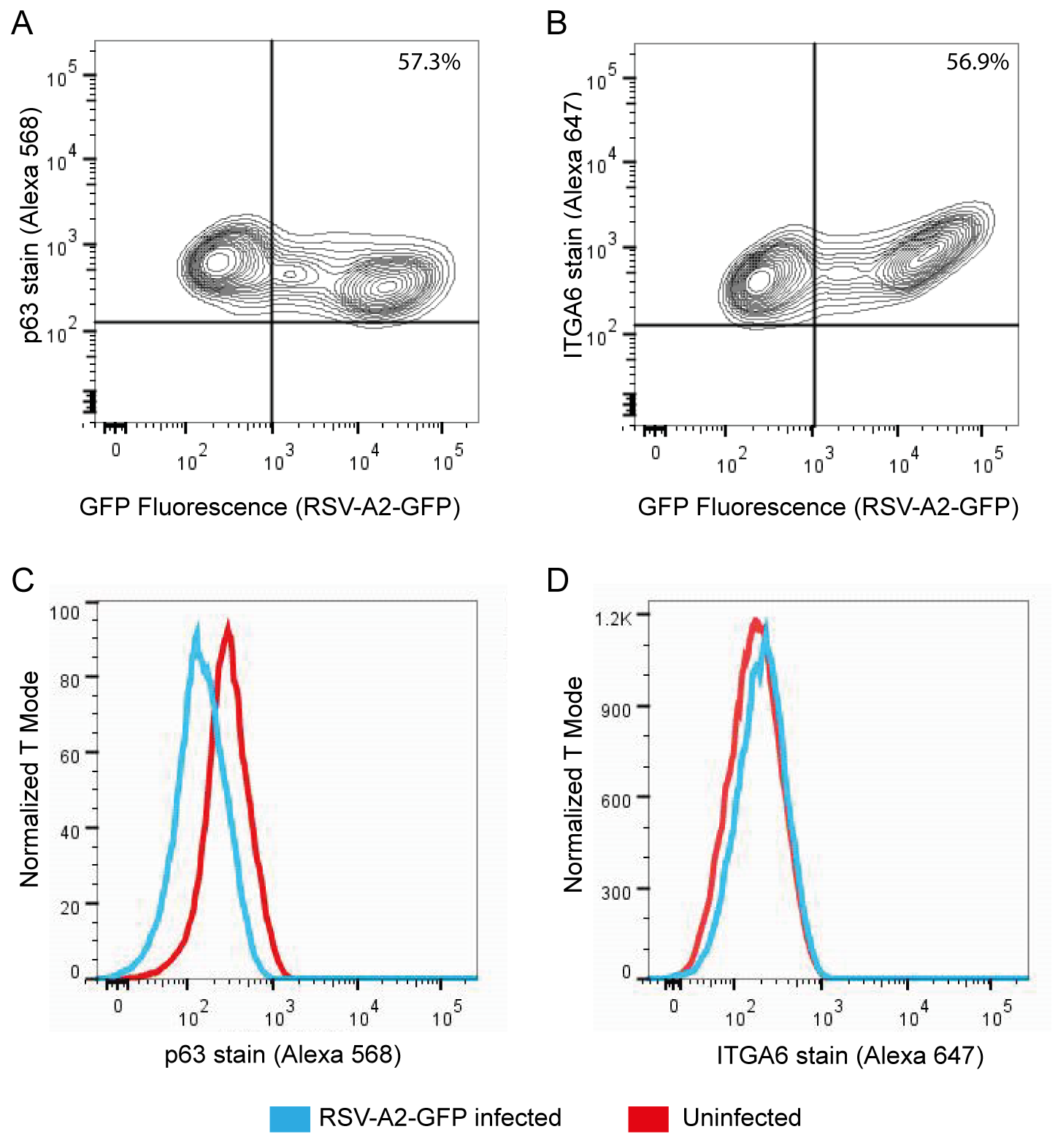

Supplement: Figure S1 — Flow cytometry analysis of RSV-A2-GFP infected basal cells. RSV infection of HBEC cells at 3 h post seeding allowed for an extensive infection of basal cells. At 45 h post infection cells were harvested, fixed and stained for the basal cell markers p63 and ITGA6. Close to 100% of the cells on the inserts were basal cells as indicated by positive p63 and ITGA6 stain (A–B). In addition, infected cells showed a significant reduction in p63 levels but not ITGA6 (C–D). Presented are representative flow cytometry data from three independent donors. (TIF) [file pone.0102368.s001.tif]

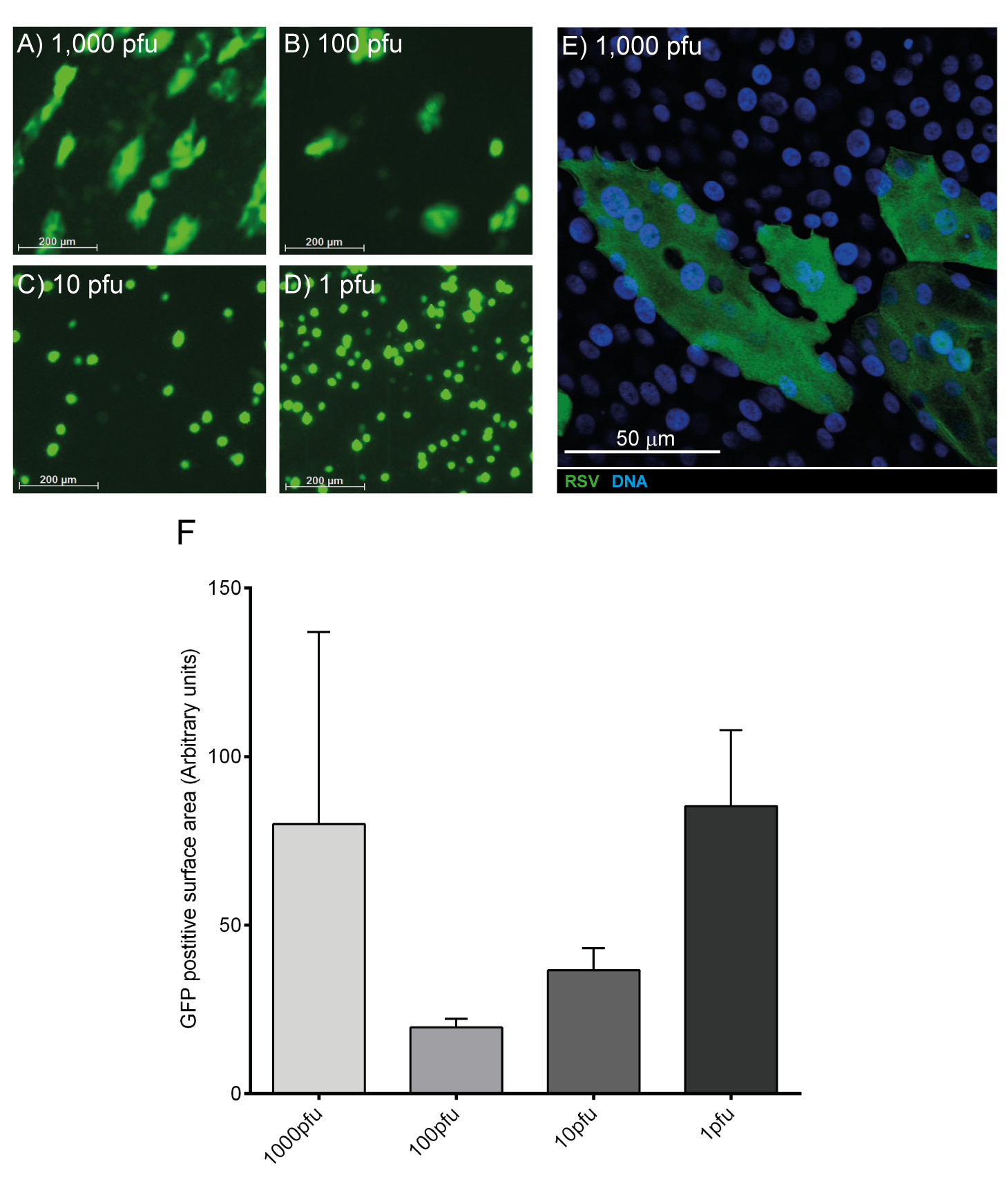

Supplement: Figure S2 — RSV-A2-GFP infection 21 days post infection. The effect of RSV-A2-GFP infection of basal cells (1–1,000 pfu/Transwell, D to A respectively, 3 h after seeding) was investigated over the duration of epithelial growth and differentiation by fluorescence imaging. Lower viral doses (C–D) exclusively generated infected cells with a condensed-morphology that gradually disappeared as the viral dose increased (A–D). The highest viral dose studied (1,000 pfu) resulted in the formation of an epithelium with large patches of infection (A, GFP+ cells) that were confirmed as syncytia by confocal imaging (E). Representative images from three independent experiments are shown, RSV infected cells are colored green. (TIF) [file pone.0102368.s002.tif]

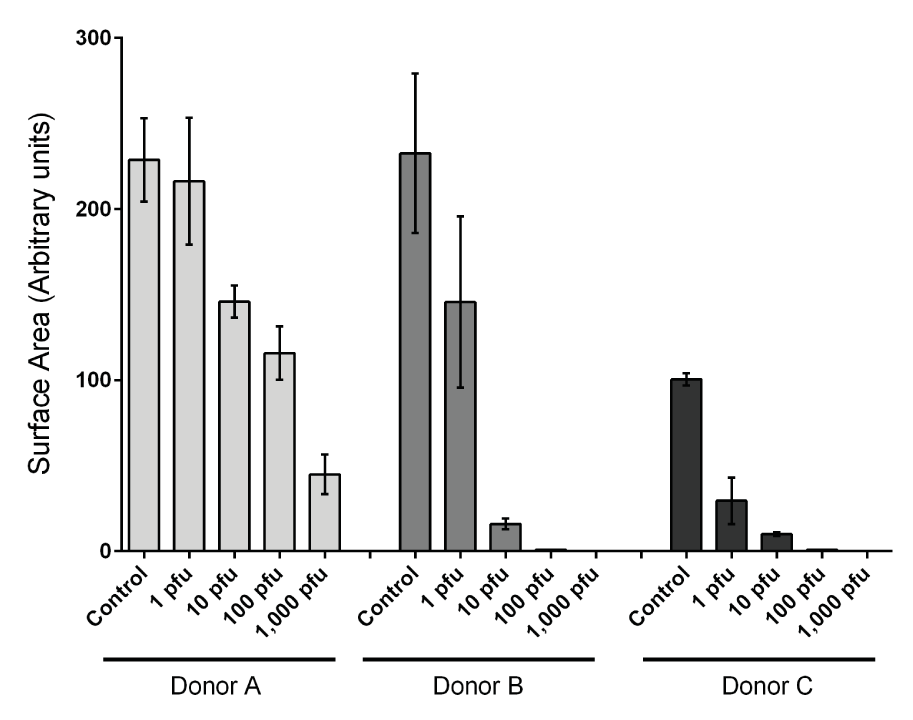

Supplement: Figure S3 — RSV induced epithelium phenotype was independent of donor. HBEC cells from three different donors were investigated to rule out any for donor variability in the loss of cilia phenotype. Cells were infected at a Transwell insert 3 h after seeding using a range of RSV-A2-GFP from 1–1000 pfu/Transwell. After 21 days in culture the cells were stained for cilia using acetylated α-tubulin. Cells from all donors were cultured and imaged in parallel and three inserts from each donor was examined. Data is presented as the average ± SD, from 2 independent experiments and a total of 4–6 inserts per viral dose. (TIF) [file pone.0102368.s003.tif]

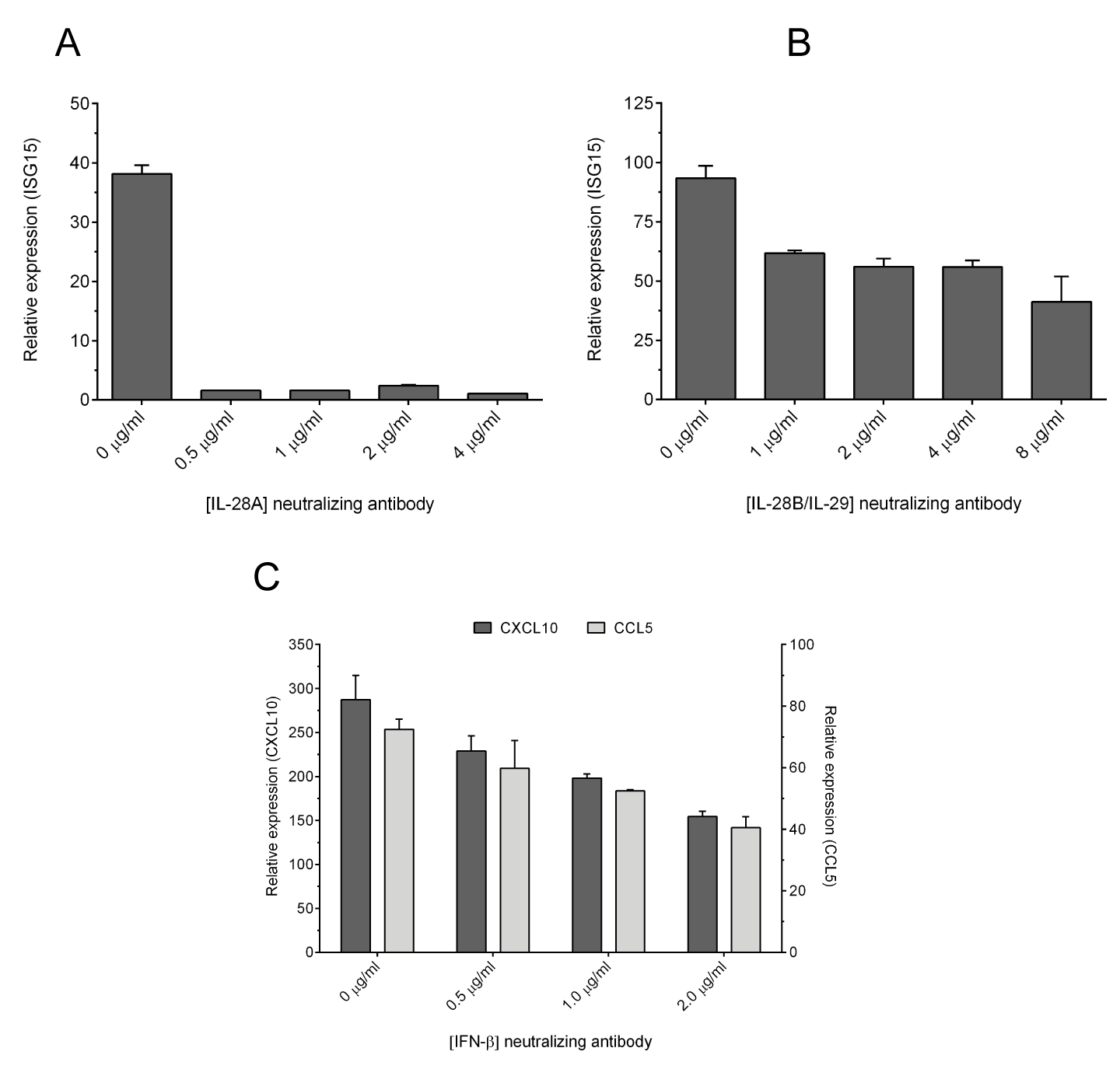

Supplement: Figure S4 — Validation of neutralizing activity of anti-interferon antibodies. Antibodies intended to neutralize IL-28A, IL-28B, IL-29 and IFN-β were all verified to neutralize a stimulated response in A549 cells. A549 cells were seeded into a 12 well plate (1.2×10^5^ cells/well) and stimulated for 2 h using 10 ng/mL of IL-28A (R & D Systems), IL-28B (R & D Systems) or IFN-β (pbl bioscience). The increasing concentrations of neutralizing antibodies added were based on the manufacturer’s neutralization data. After 24 h of treatment total RNA was collected using Buffer AVL from the RNAeasy kit (Qiagen) and RNA purified according to the manufacturer’s instructions. qRT-PCR was performed using 40 ng of total cDNA analyzing ISG15 for IL-28A/IL-28B/IL-29 stimulation (A–B) and CXCL10 for IFN-β stimulation (C). The concentration of antibody that resulted in a >50% pathway inhibition was used in the experiments presented in Figure 6. (TIF) [file pone.0102368.s004.tif]
